# Supplementary material for: scTab: Scaling cross-tissue single-cell annotation models
Source: Nat Commun. 2024 Aug 4;15:6611. doi: 10.1038/s41467-024-51059-5 (PMC11298532; doi:10.1038/s41467-024-51059-5)
Supplement: Supplementary file 1 — Supplementary Information [file 41467_2024_51059_MOESM1_ESM.pdf]

Supplementary Information

Supp. Figure 1: Comparison of scTab performance versus single-cell foundation models (scGPT and UCE).

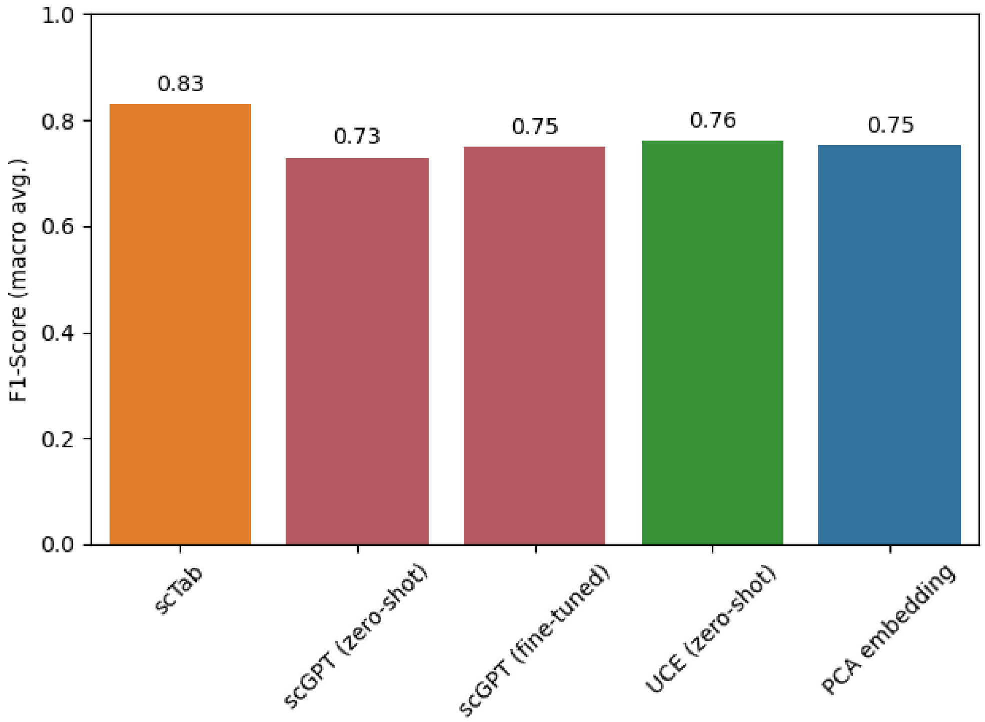

Comparison of classification performance (measured by macro F1-score) of scTab, scGPT (evaluated in zero-shot setting and fine-tuned), and Universal Cell Embedding (UCE) on the holdout test data. Additionally, the performance of a linear classifier trained on 256-dimensional PCA embeddings is shown as a reference (the linear classifier is trained on the same 1,500,000 cells that were used to train the scGPT zero-shot classifier). The fine-tuned scGPT model was fitted on 150,000 cells.Supp. Figure 2: Detailed evaluation of the classification performance of scTab and the optimized linear model. Source data are provided as a Source Data file.

Supp. Figure 2: Detailed evaluation of the classification performance of scTab and the optimized linear model.

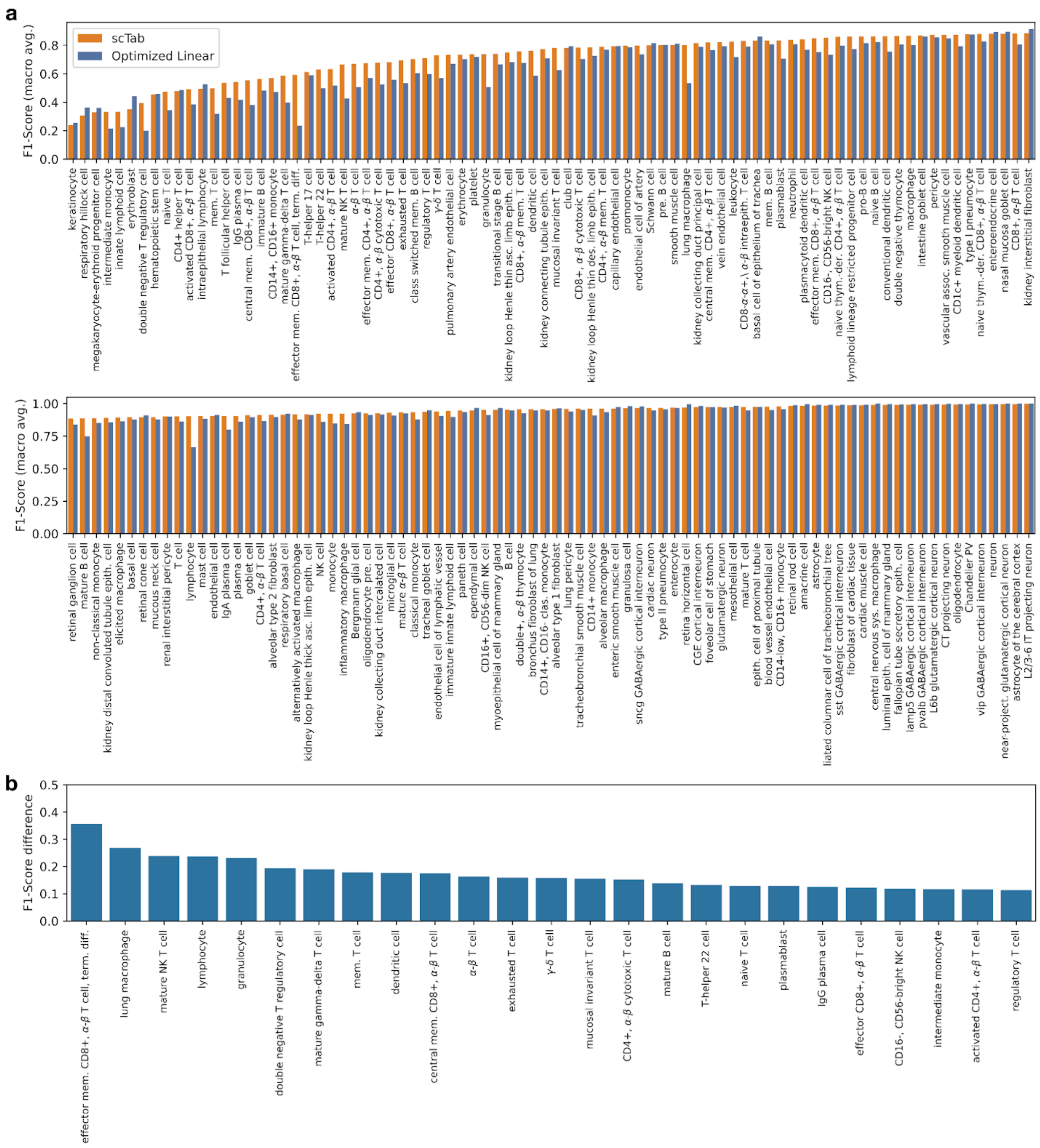

(a) Per cell type classification performance of scTab and the optimized linear model. Source data are provided as a Source Data file. **(b)** Top 25 cell types with the biggest improvement in F1-score of scTab over the optimized linear reference model. Source data are provided as a Source Data file.

Supp. Figure 3: Comparison of the classification performance of scTab and the optimized linear model in two scenarios: i) Identifying cells that belong to a coarse cell type label ii) distinguishing between fine-grained subtypes of the respective coarse cell type label (measured by the macro-averaged F1-score).

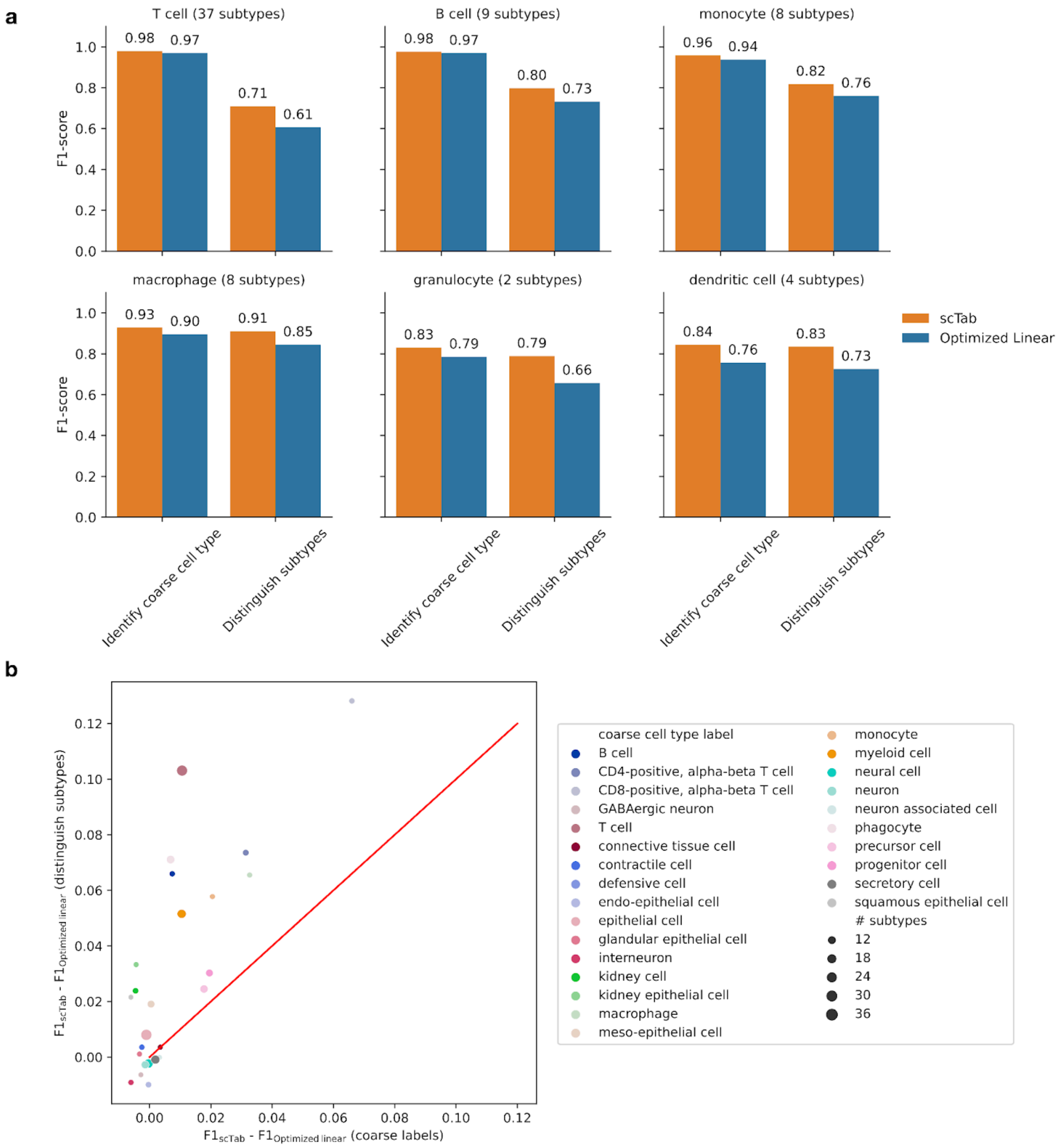

(a) Evaluation on selected coarse cell type labels of the immune system: T cells, B cells, monocytes, macrophages, granulocytes, and dendritic cells. The number of fine-grained cell type labels corresponding to a coarse cell type label is indicated beside the cell type name. Source data are provided as a Source Data file. **(b)** Systematic evaluation on cell types across the CELLxGENE data corpus. The plot shows the difference in F1-score between the scTab model and the optimized linear model on the coarse labels plotted on the x-axis and the difference in F1-score on the fine-grained annotations plotted on the y-axis in the barplot below. Additionally, shows a red reference line: For all the points above the red reference line, the performance improvement of

scTab compared to the optimized linear model increased when evaluating the classification performance on the fine-grained cell type labels. Source data are provided as a Source Data file.

Supp. Figure 4: Uncertainty scores superimposed on tSNE plot of normalized gene expression on holdout test data.

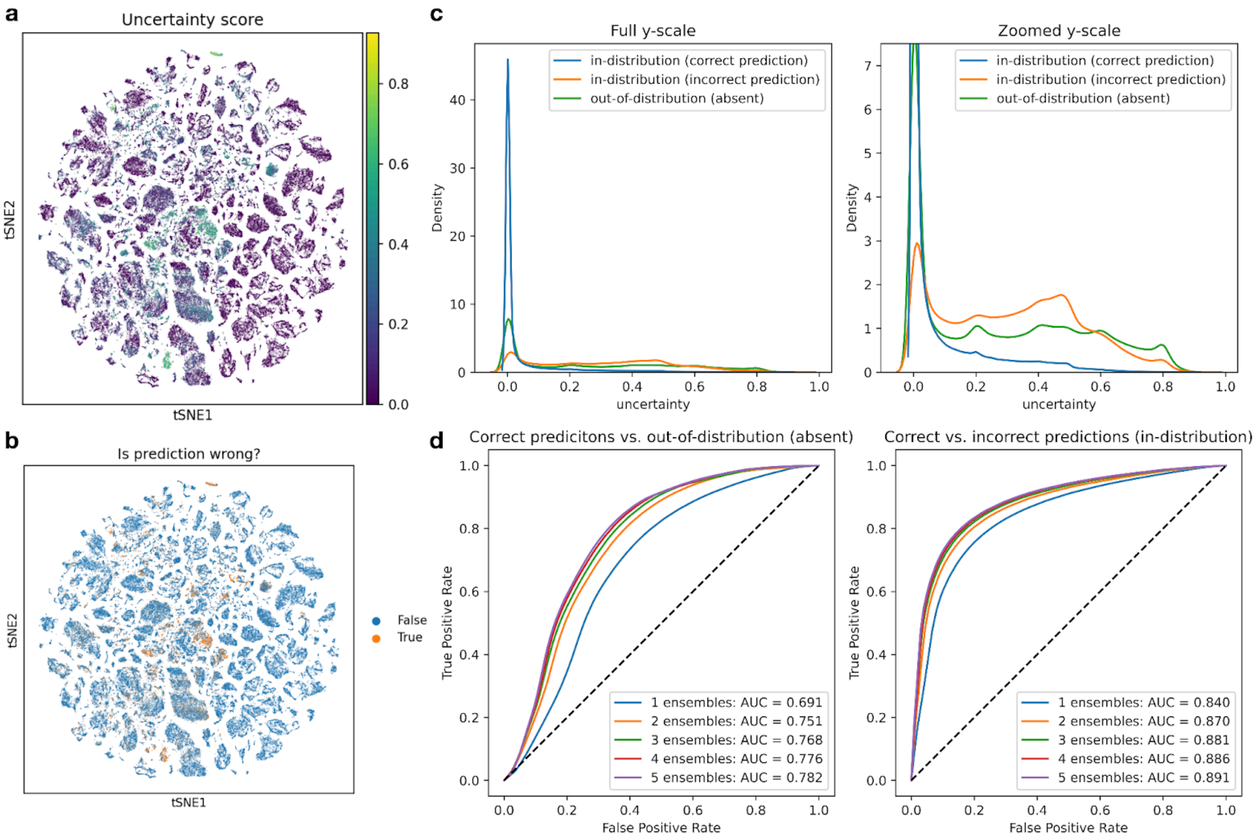

Uncertainty scores are calculated based on deep ensembles (averaged over 5 models) using $1 - maximum\, predicted\, probability$ to estimate the model uncertainty. Figures a) and b) show tSNE embeddings of the normalized gene expression on the holdout test data. **(a)** Predicted uncertainty scores. **(b)** Binary indicator of whether a prediction was wrong to visually correlate uncertainty scores with wrong predictions. **(c)** Distribution of the uncertainty scores of the scTab model split into three distinct groups: correct predictions, incorrect predictions and absent (novel cell types that are not present in the training data). **(d)** Separation of correct predictions from absent cell types (left plot) and separation of correct predictions from incorrect predictions (right plot) based on the receiver operator characteristic (ROC) curve. The plot shows the ROC curves for ensemble models consisting of one to five ensemble models. Moreover, the area under the ROC curve (AUC-ROC) for each ensemble is reported in the legend.

Supp. Figure 5: Extended data summary statistics for scTab data corpus.

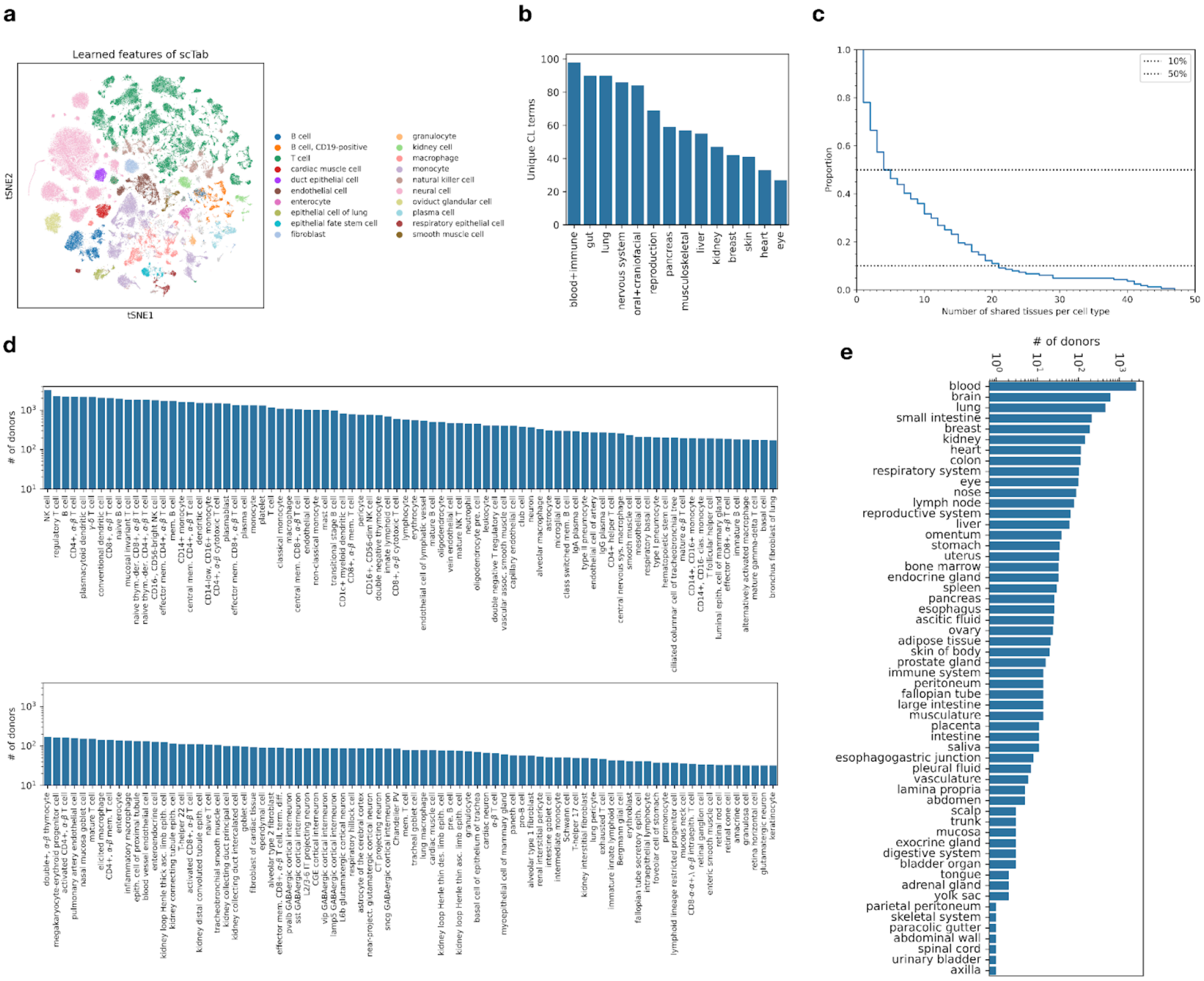

(a) Learned features of scTab on holdout test data with granular cell type labels superimposed. **(b)** Number of unique cell types (CL terms) per Human Cell Atlas bionetwork. **(c)** Number of shared tissues across individual cell types: The plot shows the complementary empirical cumulative distribution function of how many tissues each cell type is observed over (see Supp Table 7 for a per cell type statistic). **(d)** Number of unique donors per cell type. **(e)** Number of unique donors per tissue.

Supp. Figure 6: Distribution of separation scores (measured by the area under precision-recall-curve) grouped by easy-to-predict and hard-to-predict cell types.

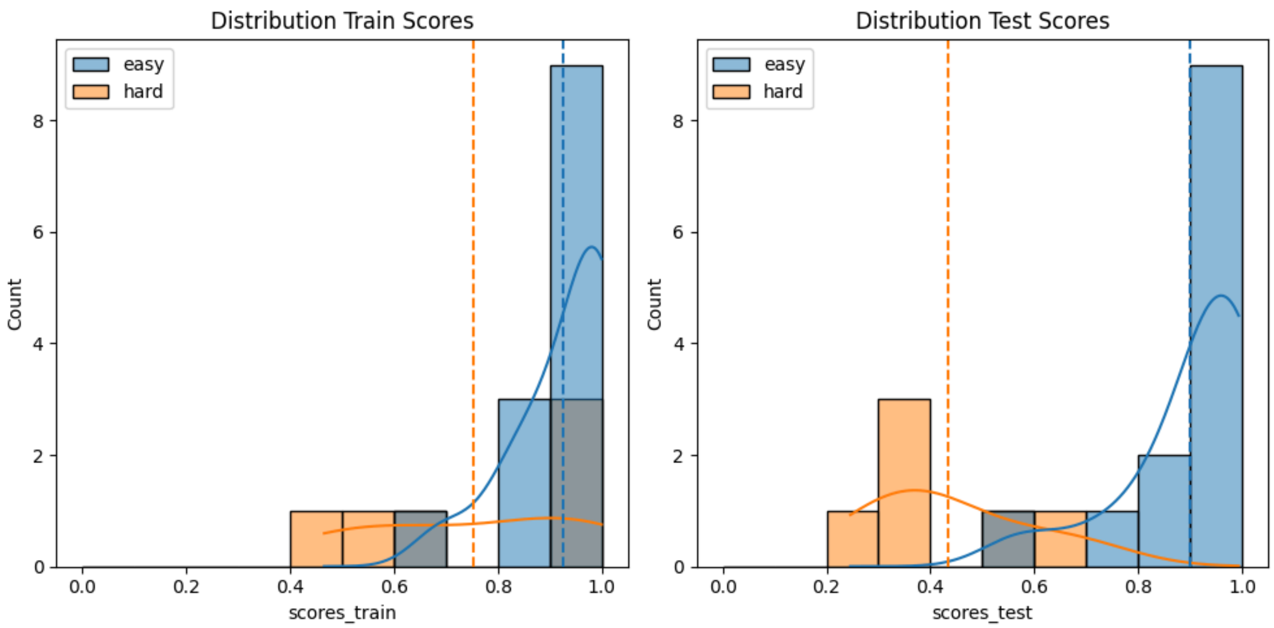

Linear classifier trained on top 200 genes obtained for each cell type obtained by looking at the scTab attention scores. The linear classifier is fit on the training data and then evaluated on the test data. The vertical dashed lines indicate the mean score per group.

Supp. Figure 7: Extended classification performance evaluation of scTab

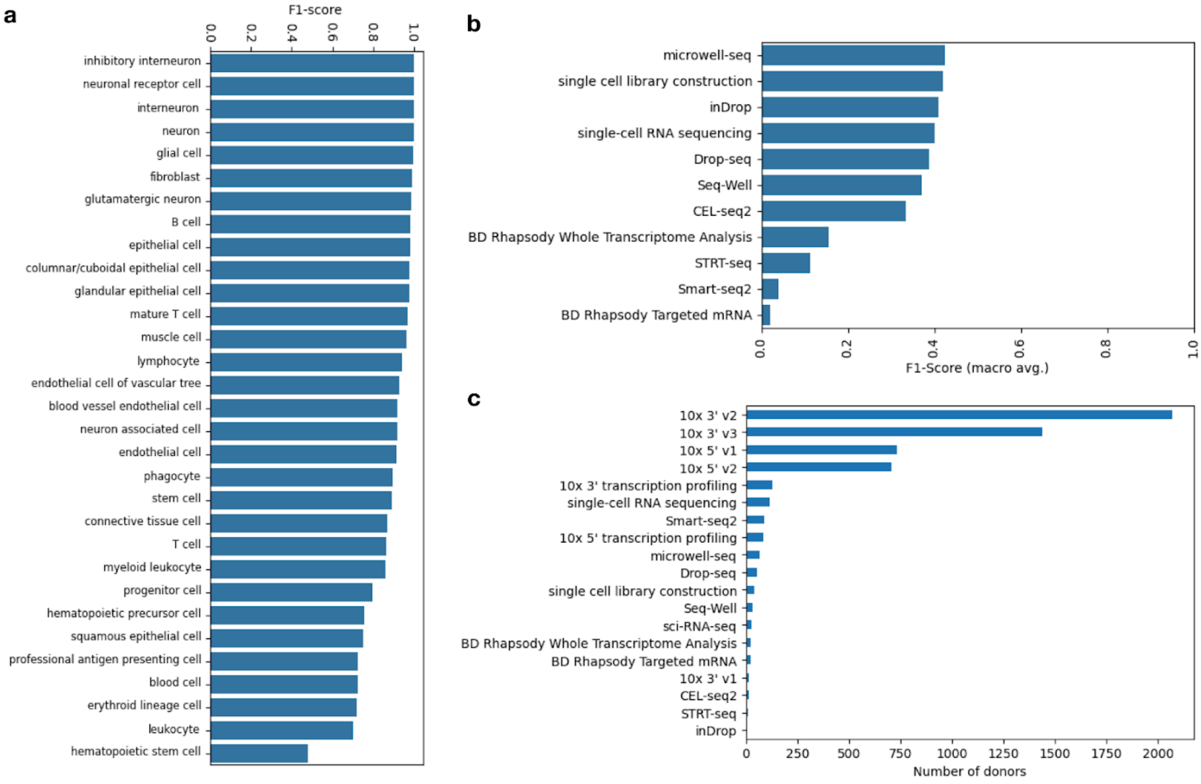

(a) Classification performance of scTab on coarse cell type labels. See Methods section for a detailed explanation of how the coarse cell types are defined. Source data are provided as a Source Data file. **(b)** Performance of our scTab model (trained only on data from 10X-based sequencing protocols) on non-10X-based data from CELLxGENE grouped by sequencing protocol. Source data are provided as a Source Data file. **(c)** The number of unique donors for each sequencing protocol in the CELLxGENE data corpus. The majority of the CELLxGENE data comes from 10X-based sequencing protocols.

Supp. Figure 8: Data loading performance during model training (with data shuffling) and inference (without data shuffling).

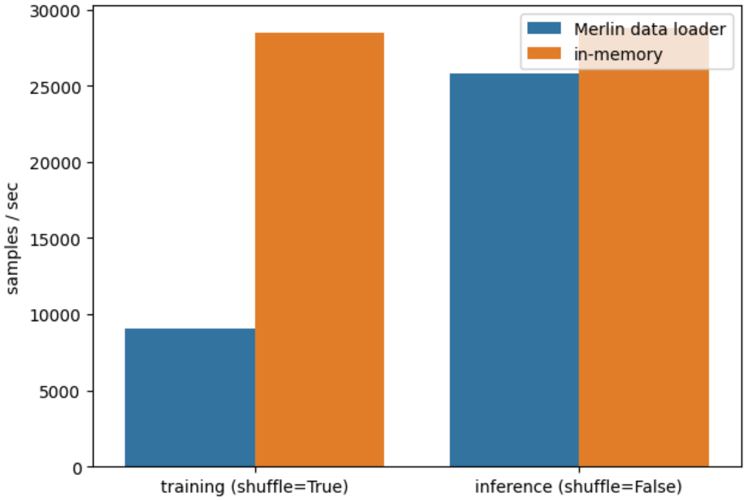

Benchmarks were run on a DGX-A100-320GB compute node with 14 cores and 80GB of memory allocated for the benchmark and half an A100 GPU (4g.20gb MIG). The training dataset consists of 15.2 million cells for the Merlin data loader and 1 million cells for the in-memory data loader. The validation dataset consists of 3.5 million cells for the Merlin data loader and 1 million cells for the in-memory data loader. Due to memory limitations for the in-memory data-loading, the training and validation set is subsampled to 1 million cells.

Supp. Figure 9: Cell type dependency of augmentation vectors before and after K-means filtering step.

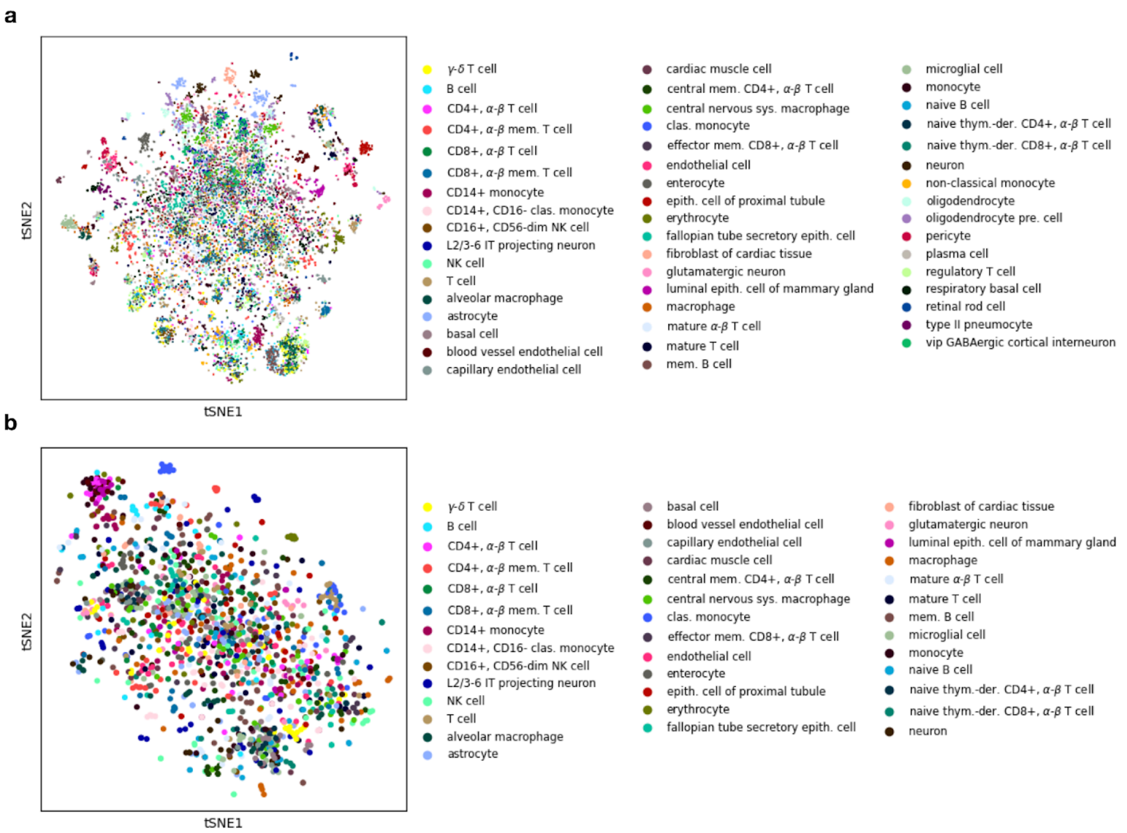

tSNE plot of augmentation vectors with the cell type based on which the augmentation vector is calculated superimposed. **(a)** Augmentation vectors before filtering for cell type independence. **(b)** Augmentation vectors after filtering for cell type independence.

Supp. Table 1: Classification performance of different models.

(a) Confidence intervals calculated over differently seeded random model initializations:

	F1-score (macro avg.)	Number of runs to calculate standard deviation
scTab (deep learning)	0.8295 ± 0.0007	5
XGBoost (boosted decision trees)	0.8127 ± 0.0005	5
MLP (deep learning)	0.7971 ± 0.0012	5
Linear	0.7848 ± 0.0001	4
CellTypist (training data subsampled to 1.5 Mio cells)	0.7304 ± 0.0015	4
scGPT (zero-shot setting)	0.7301±0.0035	5
scGPT (fine-tuned on 150,000 cells)	0.749	1
CIForm (subsampled to 750.000 cells)	0.7660	1

UCE (zero-shot setting)	0.7611±0.0018	4

(b) Confidence intervals calculated over differently seeded random model initializations and additionally including bootstrapping across donors in the test data:

	**F1-score (macro avg.)**	**Number of runs to calculate standard deviation**
scTab (deep learning)	0.8300 ±0.0069	4
XGBoost (boosted decision trees)	0.8136 ± 0.0060	4
MLP (deep learning)	0.7973 ± 0.0074	4
Linear	0.7846 ± 0.0072	4
CellTypist (training data subsampled to 1.5 Mio cells)	0.7291 ± 0.0072	4

Supp. Table 2: Training time and inference time comparison of the models used in this paper

	**Training time [in hours]**	**Inference time [samples/sec]**
CellTypist (trained on 1.5 Mio cells)	~16h (AMD EPYC 7642 - 20 cores)	~2000 (AMD EPYC 7642 - 20 cores)
Optimized linear	~20h (1x Nvidia A100 40GB)	~29500 (1x Nvidia V100 16GB)
scTab	~33h (1x Nvidia A100 40GB)	~10800 (1x Nvidia V100 16GB)
XGBoost	~10h (1x Nvidia A100 80GB)	~4200 (1x Nvidia V100 16GB)
MLP	~29h (1x Nvidia A100 40GB)	~21400 (1x Nvidia V100 16GB)

Explanation of different hardware used:

- CellTypist does not have GPU support. Hence, we could not use a GPU to speed up training or inference.
- XGBoost has very high GPU memory requirements as it is not trained in batches, unlike the other methods. Therefore, we had to use A100 80GB to be able to train the model
- Inference benchmarks are done on V100 GPUs as more computing power was not needed for inference and they are more available on our HPC cluster.

Supp. Table 3: Easy-to-predict cell types despite little available training data

	**F1-score**	**Number of cells**
chandelier pvalb GABAergic cortical interneuron	0.993885	7268
L6b glutamatergic cortical neuron	0.9928	9727
caudal ganglionic eminence derived GABAergic cortical interneuron	0.973098	6299
retina horizontal cell	0.971226	8530
cardiac neuron	0.967201	4629

lung pericyte	0.959292	3254
bronchus fibroblast of lung	0.956621	4299
ependymal cell	0.948148	4709
paneth cell	0.946496	3328
Bergmann glial cell	0.924838	4564
inflammatory macrophage	0.923077	8629
alternatively activated macrophage	0.917476	7041
renal interstitial pericyte	0.902137	4455

Supp. Table 4: Total variation that can be attributed to the cell type before and after data augmentation

	**Total variation attributed to cell type and donor (R^2)**
original/non-augmented data	0.189
augmented data	0.164

Supp. Table 5: Effect of data augmentation on loss and F1-score (macro avg.) on holdout test set.

	**Neg. log-likelihood**	**F1-score (macro avg.)**	**Number of runs to calculate standard deviation**
w. augmentation	0.659 ± 0.04	0.7841 ± 0.0030	4
wo. augmentation	0.797 ± 0.05	0.7755 ± 0.0020	4
P-value	0.0039	0.0016	4

Supp. Table 6: Classification performance of models with tuned versus default hyperparameters.

	**F1-score (macro avg.) with default parameters**	**F1-score (macro avg.) with tuned parameters**	**Number of runs to calculate standard deviation**
XGBoost	0.5855 ± 0.0112	0.8127 ± 0.0005	4
CellTypist	0.6258 ± 0.0036	0.7304 ± 0.0015	4
