# Supplementary material for: scTab: Scaling cross-tissue single-cell annotation models
Source: Nat Commun. 2024 Aug 4;15:6611. doi: 10.1038/s41467-024-51059-5 (PMC11298532; doi:10.1038/s41467-024-51059-5)
Supplement: Supplementary file 3 — Reporting Summary [file 41467_2024_51059_MOESM3_ESM.pdf]

nature portfolio

Corresponding author(s): Prof. Fabian Theis

Last updated by author(s): Jul 5, 2024

Reporting Summary

Nature Portfolio wishes to improve the reproducibility of the work that we publish. This form provides structure for consistency and transparency in reporting. For further information on Nature Portfolio policies, see our Editorial Policies and the Editorial Policy Checklist.

Statistics

For all statistical analyses, confirm that the following items are present in the figure legend, table legend, main text, or Methods section.

n/a	Confirmed	
[ ]	[x]	The exact sample size (*n*) for each experimental group/condition, given as a discrete number and unit of measurement
[x]	[ ]	A statement on whether measurements were taken from distinct samples or whether the same sample was measured repeatedly
[x]	[ ]	The statistical test(s) used AND whether they are one- or two-sided<br>*Only common tests should be described solely by name; describe more complex techniques in the Methods section.*
[x]	[ ]	A description of all covariates tested
[x]	[ ]	A description of any assumptions or corrections, such as tests of normality and adjustment for multiple comparisons
[ ]	[x]	A full description of the statistical parameters including central tendency (e.g. means) or other basic estimates (e.g. regression coefficient) AND variation (e.g. standard deviation) or associated estimates of uncertainty (e.g. confidence intervals)
[x]	[ ]	For null hypothesis testing, the test statistic (e.g. *F*, *t*, *r*) with confidence intervals, effect sizes, degrees of freedom and *P* value noted<br>*Give P values as exact values whenever suitable.*
[x]	[ ]	For Bayesian analysis, information on the choice of priors and Markov chain Monte Carlo settings
[x]	[ ]	For hierarchical and complex designs, identification of the appropriate level for tests and full reporting of outcomes
[x]	[ ]	Estimates of effect sizes (e.g. Cohen's *d*, Pearson's *r*), indicating how they were calculated

Our web collection on statistics for biologists contains articles on many of the points above.

Software and code

Policy information about availability of computer code

Data collection	All data was collected from the CELLxGENE census version 2023-05-15. Census version 2023-05-15 is a long-term-support version and while be hosted by CELLxGENE for at least 5 years.
Data analysis	All code used for our analysis can be found under: https://github.com/theislab/scTab

For manuscripts utilizing custom algorithms or software that are central to the research but not yet described in published literature, software must be made available to editors and reviewers. We strongly encourage code deposition in a community repository (e.g. GitHub). See the Nature Portfolio guidelines for submitting code & software for further information.

Data

Policy information about availability of data

All manuscripts must include a data availability statement. This statement should provide the following information, where applicable:

- Accession codes, unique identifiers, or web links for publicly available datasets
- A description of any restrictions on data availability
- For clinical datasets or third party data, please ensure that the statement adheres to our policy

GitHub - All code: https://github.com/theislab/scTab
GitHub - Tutorials:
* Data loading tutorial: https://github.com/theislab/scTab/blob/main/notebooks-tutorials/data_loading.ipynb

* Loading trained models: https://github.com/theislab/scTab/blob/main/notebooks-tutorials/model_inference.ipynb

Data: https://pklab.med.harvard.edu/felix/data/merlin_cxg_2023_05_15_sf-log1p.tar.gz (164GB)
Checkpoints: https://pklab.med.harvard.edu/felix/data/scTab-checkpoints.tar.gz (8.1GB)

Research involving human participants, their data, or biological material

Policy information about studies with human participants or human data. See also policy information about sex, gender (identity/presentation), and sexual orientation and race, ethnicity and racism.

Reporting on sex and gender	n/a
Reporting on race, ethnicity, or other socially relevant groupings	n/a
Population characteristics	n/a
Recruitment	n/a
Ethics oversight	n/a

Note that full information on the approval of the study protocol must also be provided in the manuscript.

Field-specific reporting

Please select the one below that is the best fit for your research. If you are not sure, read the appropriate sections before making your selection.

[x] Life sciences [ ] Behavioural & social sciences [ ] Ecological, evolutionary & environmental sciences

For a reference copy of the document with all sections, see nature.com/documents/nr-reporting-summary-flat.pdf

Life sciences study design

All studies must disclose on these points even when the disclosure is negative.

Sample size	The sample size was limited by the current size of the human CELLxGENE census.
Data exclusions	The following criteria are used to filter the human CELLxGENE census data:<br><br>1. The census data is subset to primary data only (is_primary_data == True) to prevent label leakage between the train, validation, and test set.<br>2. Only sequencing data from 10x-based sequencing protocols is used. In terms of the CELLxGENE census, this means subsetting the assay metadata column to the following terms: 10x 5’ v2, 10x 3’ v3, 10x 3’ v2, 10x 5’ v1, 10x 3’ v1, 10x 3’ transcription profiling, 10x 5’ transcription profiling<br>3. The annotated cell type has to be a subtype of the native cell label based on the underlying cell type ontology<br>4. For each cell type, there have to be at least 5,000 unique cells. Otherwise, the whole cell type is dropped from the dataset.<br>5. Each cell type has to be observed across at least 30 donors to reliably quantify whether the trained classifier can generalize to new unseen donors for each cell type. With the used 70-15-15 train, validation, and test split this means that each cell type is represented with at least 4-5 donors in the validation and test set, respectively.<br>6. Each cell type needs to have at least seven parent nodes in the cell type ontology. This criterion is used as a heuristic to filter out general cell type labels that do not contain much information.
Replication	The splits into training, validation and test sets are performed with fixed random seeds to reproduce our training and evaluation data.<br>All models were fitted several times with different random seeds to estimate the standard deviation of the model performances.
Randomization	Splitting into training, validation and test set was done based on random selection.
Blinding	n/a, as we didn't do any data collection ourselves, we just used the data from CELLxGENE census.

Reporting for specific materials, systems and methods

We require information from authors about some types of materials, experimental systems and methods used in many studies. Here, indicate whether each material, system or method listed is relevant to your study. If you are not sure if a list item applies to your research, read the appropriate section before selecting a response.

Materials & experimental systems

n/a	Involved in the study
☒	☐ Antibodies
☒	☐ Eukaryotic cell lines
☒	☐ Palaeontology and archaeology
☒	☐ Animals and other organisms
☒	☐ Clinical data
☒	☐ Dual use research of concern
☒	☐ Plants

Methods

n/a	Involved in the study
☒	☐ ChIP-seq
☒	☐ Flow cytometry
☒	☐ MRI-based neuroimaging

Plants

Seed stocks	n/a
Novel plant genotypes	n/a
Authentication	n/a
